# Supplementary material for: Polyketide synthase gene clusters in the ericoid mycorrhizal fungus Oidiodendron maius: genome-wide prediction, expression profiling, and functional characterization of knockout mutants
Source: Front Plant Sci. 2026 Sep 16;17:1924430. doi: 10.3389/fpls.2026.1924430 (PMC13623903; doi:10.3389/fpls.2026.1924430)
Supplement: Supplementary file 1 [file SupplementaryFile1.zip › Supplementary Materials/Supplementary Folder 1.zip!/Oidma1_AssemblyScaffolds_Repeatmasked/knownclusterblast/cluster21/jgi.p_Oidma1_42472_mibig_hits.html]

| MIBiG Protein | Description | MIBiG Cluster | MiBiG Product | % ID | % Coverage | BLAST Score | E-value |
| --- | --- | --- | --- | --- | --- | --- | --- |
| BAE56591.1 |  | BGC0001123 | Aspirochlorine\_biosynthetic\_gene\_cluster | 42 | 96.015936255 | 380 | 2.6e-105 |
| OAQ83755.1 | isotrichodermin\_C-15\_hydroxylase | BGC0001358 | Leucinostatins\_biosynthetic\_gene\_cluster | 40 | 98.8047808765 | 352 | 9.9e-97 |
| BAK26559.1 | cytochrome\_P450 | BGC0000977 | Cyclopiazonic\_acid\_biosynthetic\_gene\_cluster | 39 | 95.219123506 | 334 | 2.8e-91 |
| AAS90101.1 | AvnA | BGC0000006 | Aflatoxin\_biosynthetic\_gene\_cluster | 40 | 94.6215139442 | 328 | 1.2e-89 |
| BAE71325.1 | cytochrome\_P450\_monooxygenase | BGC0000004 | Aflatoxin\_biosynthetic\_gene\_cluster | 40 | 94.6215139442 | 328 | 1.2e-89 |
| AAS90032.1 | AvnA | BGC0000008 | Aflatoxin\_biosynthetic\_gene\_cluster | 40 | 94.6215139442 | 327 | 2e-89 |
| AAK53580.1 | isotrichodermin\_C-15\_hydroxylase | BGC0000930 | Trichothecene\_biosynthetic\_gene\_cluster | 37 | 99.203187251 | 327 | 2.6e-89 |
| AAS90078.1 | AvnA | BGC0000010 | Aflatoxin\_biosynthetic\_gene\_cluster | 40 | 94.6215139442 | 326 | 4.5e-89 |
| AAK33070.1 | trichothecene\_C-15\_hydroxylase | BGC0001278 | Nivalenol\_/\_deoxynivalenol\_/\_3-acetyldeoxynivalenol\_/ | 38 | 98.2071713147 | 325 | 1e-88 |
| AAK33080.1 | putative\_trichothecene\_C-15\_hydroxylase | BGC0001277 | Nivalenol\_/\_deoxynivalenol\_/\_3-acetyldeoxynivalenol\_/ | 38 | 99.8007968127 | 324 | 2.9e-88 |
| AAK53587.1 | isotrichodermin\_C-15\_hydroxylase | BGC0000931 | Trichothecene\_biosynthetic\_gene\_cluster | 38 | 99.8007968127 | 323 | 4.9e-88 |
| AAS90058.1 | AvnA | BGC0000009 | Aflatoxin\_biosynthetic\_gene\_cluster | 40 | 94.2231075697 | 319 | 5.5e-87 |
| AAS90010.1 | AvnA | BGC0000007 | Aflatoxin\_biosynthetic\_gene\_cluster | 39 | 94.2231075697 | 317 | 3.5e-86 |
| ACH72905.1 | AflG | BGC0000011 | Aflatoxin/sterigmatocystin\_biosynthetic\_gene\_cluster | 38 | 97.0119521912 | 316 | 4.6e-86 |
| AAC49196.1 | putative\_p450\_monooxygenase | BGC0000152 | Sterigmatocystin\_biosynthetic\_gene\_cluster | 39 | 89.2430278884 | 315 | 1.3e-85 |
| ACD39760.1 | cytochrome\_P450 | BGC0000077 | Hypothemycin\_biosynthetic\_gene\_cluster | 36 | 96.4143426295 | 304 | 2.4e-82 |
| ACB12551.1 | Fum2 | BGC0000063 | Fumonisin\_biosynthetic\_gene\_cluster | 35 | 97.609561753 | 304 | 2.4e-82 |
| ACD39751.1 | cytochrome\_P450 | BGC0000076 | Hypothemycin\_biosynthetic\_gene\_cluster | 36 | 96.4143426295 | 303 | 5.3e-82 |
| AAN74815.2 | Fum2p | BGC0000062 | Fumonisin\_biosynthetic\_gene\_cluster | 36 | 97.4103585657 | 299 | 7.6e-81 |
| AAM18769.1 | AflL | BGC0000011 | Aflatoxin/sterigmatocystin\_biosynthetic\_gene\_cluster | 38 | 97.8087649402 | 298 | 1.3e-80 |
| BAD83682.1 | cytochrome\_P-450 | BGC0000012 | Alternapyrone\_biosynthetic\_gene\_cluster | 35 | 95.0199203187 | 287 | 3.9e-77 |
| Bcin12g06370.1 |  | BGC0000631 | Botrydial\_biosynthetic\_gene\_cluster | 35 | 93.0278884462 | 283 | 5.7e-76 |
| BAQ25465.1 | cytochrome\_P450 | BGC0001280 | Betaenone\_C\_/\_betaenone\_A\_biosynthetic\_gene\_cluster | 36 | 95.6175298805 | 280 | 2.8e-75 |
| BAD29972.1 | P450\_monooxygenase\_1 | BGC0000676 | Aphidicolin\_biosynthetic\_gene\_cluster | 36 | 98.2071713147 | 280 | 4.8e-75 |
| EFY94501.1 | benzoate\_4-monooxygenase\_cytochrome\_P450 | BGC0000337 | Destruxins\_biosynthetic\_gene\_cluster | 35 | 97.2111553785 | 273 | 5.9e-73 |
| ADI24937.1 | VrtK | BGC0000168 | Viridicatumtoxin\_biosynthetic\_gene\_cluster | 34 | 102.988047809 | 270 | 5e-72 |
| EAU36742.1 | predicted\_protein | BGC0000292 | Acetylaranotin\_biosynthetic\_gene\_cluster | 28 | 40.438247012 | 78 | 2.1e-14 |
| AAC49192.1 | putative\_p450\_monooxygenase | BGC0000152 | Sterigmatocystin\_biosynthetic\_gene\_cluster | 29 | 35.8565737052 | 74 | 5.2e-13 |
| ABI22130.1 | cytochrome\_P450\_hydroxylase | BGC0000422 | Saframycin\_A\_biosynthetic\_gene\_cluster | 31 | 22.1115537849 | 64 | 5.4e-10 |
| EAU36745.1 | hypothetical\_protein | BGC0000292 | Acetylaranotin\_biosynthetic\_gene\_cluster | 28 | 40.0398406375 | 62 | 1.2e-09 |
| BAU61568.1 | putative\_cytochrome\_P450 | BGC0001375 | Penitrem\_biosynthetic\_gene\_cluster | 26 | 38.4462151394 | 59 | 1e-08 |
